# Editorial: Women in psychiatry 2021: Neuroimaging and stimulation

**DOI:** 10.3389/fpsyt.2022.1057330

**Published:** 2022-10-18

**Authors:** Neeltje E. M. van Haren, Martine Hoogman

**Affiliations:** ^1^Department of Child and Adolescent Psychiatry/Psychology, Erasmus Medical Centre – Sophia Children's Hospital, Rotterdam, Netherlands; ^2^Department of Psychiatry, Radboud University Medical Center, Nijmegen, Netherlands; ^3^Department of Human Genetics, Radboud University Medical Center, Nijmegen, Netherlands; ^4^Donders Institute for Brain, Cognition and Behavior, Radboud University, Nijmegen, Netherlands

**Keywords:** neuroimaging, psychiatric disorders, women scientists, brain stimulation, talents

With the Research Topic series *Women in Psychiatry*, Frontiers in Psychiatry offers a unique platform to promote the work of female scientists across all fields of Psychiatry. It thereby increases visibility of female scientists by showcasing the depth of talent and the excellence and innovativeness of their work. We are proud to showcase in this Research Topic *Women in Psychiatry – Neuroimaging and Stimulation nine* excellent contributions to the field, all first and/or last-authored by our female colleagues.

Studies were performed across a wide variety of populations, ranging from healthy individuals to individuals with psychiatric disorders, and across different neuroimaging and brain stimulation methods.

Two papers contribute to understanding healthy brain development. van Aalst et al. conducted a yoga intervention in healthy young adult females and showed effect on behavioral but not on multimodal imaging biomarkers. Blok et al. applied normative modeling in a large longitudinal dataset of T1-weighted images from the Generation R population study to establish typical development curves for (sub-)cortical volume and cortical thickness and showed how trajectories deviate in the presence of psychopathological symptoms.

Several papers contribute to advancing our understanding of the effect of treatment on outcome and symptoms of psychiatric disorders. Kahn et al. describe the effect of deep brain stimulation in patients with obsessive-compulsive disorder and psychiatric comorbidity while Baumann et al. report the findings of the effect of Transcranial Direct Current Stimulation in anorexia nervosa. Moreover, the effects of lithium response were investigated on amygdala and hippocampal shape using 7T MRI in bipolar disorder by Athey et al.

Two contributions aimed to understand the underlying biological mechanisms of psychiatric disorders. Jakobi et al. studied the neural correlates of observing dynamic facial expressions with levels of reactive aggression in adult attention-deficit/hyperactivity disorder using fMRI. Proteau-Lemieux et al. set out to investigate whether EEG markers of brain maturation are affected in fragile X syndrome.

Finally, in addition to empirical studies, the Research Topic contains a perspective and a review. Conelea et al. wrote a perspective article on the need to combine cognitive behavioral therapies (CBT) and non-invasive brain stimulation (NIBS) with the aim of improving the treatment of psychiatric disorders while Cubillo integrated the literature on the neurobiological correlates of the social and emotional impact of peer victimization.

This Research Topic celebrates the breadth of scientific ideas, techniques, approaches, and findings that female scientists contribute to the field of psychiatric neuroimaging and stimulation. It is a demonstration of creativity, vision, and perseverance. We hope it serves as an inspiration for neuroscientists of all ages, genders, cultures, or socioeconomic backgrounds.

## Author contributions

NH wrote the manuscript. MH critically reviewed the manuscript. All authors approved the final version.

## Conflict of interest

The authors declare that the research was conducted in the absence of any commercial or financial relationships that could be construed as a potential conflict of interest.

## Publisher's note

All claims expressed in this article are solely those of the authors and do not necessarily represent those of their affiliated organizations, or those of the publisher, the editors and the reviewers. Any product that may be evaluated in this article, or claim that may be made by its manufacturer, is not guaranteed or endorsed by the publisher.

